# Exosomal circular RNA hsa_circ_007293 promotes proliferation, migration, invasion, and epithelial–mesenchymal transition of papillary thyroid carcinoma cells through regulation of the microRNA-653-5p/paired box 6 axis

**DOI:** 10.1080/21655979.2021.2000745

**Published:** 2021-12-06

**Authors:** Qiuyu Lin, Qianle Qi, Sen Hou, Zhen Chen, Nan Jiang, Laney Zhang, Chenghe Lin

**Affiliations:** a Nuclear Medicine Department, The First Hospital of Jilin University, Changchun, China; b Chengdu Xinke Pharmaceutical Co., LTD, Chengdu, China; c College of Biological Sciences, Cornell University, Ithaca, NY, USA

**Keywords:** Papillary thyroid carcinoma, exosomes, circular RNAs, epithelial–mesenchymal transition, paired box 6

## Abstract

Circular RNAs (circRNAs) or exosomes have been reported to exert key regulatory and/or communication functions in human cancer. Nevertheless, current literature on the effects of exosomal circRNAs on tumor invasion and metastasis in thyroid cancer is incomplete. The role of tumor-derived exosomes in driving *in vitro* papillary thyroid carcinoma (PTC) progression and metastasis requires further investigation. In our study, Exosomes were harvested from PTC patient serum and PTC cell culture medium. Gene expression analysis in PTC cell lines and exosomes was performed with quantitative reverse-transcription polymerase chain reaction. Transwell, wound healing, Western blot assays, and the cell counting kit-8 were applied for functional analysis. Dual-luciferase reporter assay was used to examine the interaction between hsa_circ_007293 (circ007293), microRNA (miR)-653-5p, and paired box 6 (PAX6). Results showed that circ007293 was enriched in exosomes derived from PTC patient serum and cell culture media. Moreover, circ007293 could enter PTC cells through exosomes, and exosomal circ007293 promoted PTC cell epithelial–mesenchymal transition, invasion, migration, and proliferation. circ007293 knockdown reversed the malignant phenotype of PTC cells *in vitro*. Additionally, circ007293 could competitively bind with miR-653-5p to regulate PAX6 expression. Notably, miR-653-5p overexpression or PAX6 inhibition suppressed the malignant effects of exosomal circ007293. These results evidenced that exosomal circ007293 induced EMT and augmented the invasive and migratory abilities of PTC cells via the miR-653-5p/PAX6 axis, suggesting that it may serve as a promising biomarker for cancer progression.

## Introduction

Thyroid cancer is a common type of endocrine-related tumor. 85% of all thyroid malignancies are papillary thyroid carcinomas (PTC) [1]. Increasing evidence has confirmed that PTC metastasis is largely driven by the epithelial-mesenchymal transition (EMT) [2]. In the process of EMT, cancer cells lose polarity and adhesion and acquire invasive and metastatic characteristics [3]. Hence, elucidating the underlying mechanism of PTC metastasis is crucial for the development of more advanced treatment options. Circular RNAs (circRNAs) are a recently discovered form of non-coding RNA (ncRNA) that are characterized by the lack of 5ʹ to 3ʹ polarity and a polyadenylated tail in their covalent closed loop structure [4]. circRNAs appear to significantly modulate gene expression by functioning as ‘sponges’ for microRNA (miRNAs), RNA-binding protein chelating agents, or nuclear transcription regulators [5]. Notably, several circRNAs has been reported to participate in the developemt of PTC [6]. For instance, circITCH inhibited PTC progression by acting as an miR-22-3p sponge [7] and repressed glucose uptake in melanoma by directly downregulating glucose transporter type 1 expression [8]. Nevertheless, the roles of circRNAs in PTC require further investigation.

Exosomes can influence the function of target cells by transferring bioactive molecular, including RNA, lipid and protein [9,10]. What is more, exosomal circRNA has been documented to play a crucial role in the progression of human cancers. As an example, circ_0006156 was upregulated in PTC tissues and cell lines, as well as the serum exosomes from PTC patients, indicating that circ_0006156 might be a novel biomarker for PTC [11]. Additionaly, tumor-released exosomal circ-PDE8A absorbed miR-338 to regulate metastasis associated in colon cancer 1 and promoted the invasive growth of pancreatic ductal adenocarcinoma cells via the metastasis associated in colon cancer /MET/extracellular regulated protein kinases or protein kinase B pathways [12]. Further, exosomal circRNA 0001445 servered as a sponge for miRNA-127-5p to increase sorting nexin 5 expression and then facilitate the progression of glioma [13]. Notably, using high-throughput sequencing, Yang et al. [14] found that exosomes extracted from the sera of PTC patients displayed upregulated hsa_circ_007293 (circ007293) expression. Despite these data, further research is needed to elucidate the exact role of exosomal circ007293 in PTC.

This study seeks to investigate the role of exosomal circ007293 in PTC development and clarify its underlying molecular mechanism. Our data revealed that exosomal circ007293-induced EMT and augmented the metastasis of PTC cells through regulation of miR-653-5p/paired box 6 (PAX6) axis. Our findings provide the foundation for further research on exosomes and circRNAs in PTC.

## Materials and methods

### Patient samples

Blood samples were collected from 40 PTC patients and 40 healthy controls without a prior diagnosis of cancer who were treated at the First Hospital of Jilin University. None of the participants had a history of receiving radiotherapy or chemotherapy. Written informed consent was obtained from all participants. Our study’s ethical approval was granted by the Medical Ethics Committee of The First Hospital of Jilin University.

### Cell culture

Human PTC cell lines (TPC-1 and KTC-1) were obtained from Procell (Wuhan, China). The human thyroid follicular epithelial cell line (Nthy-ori3-1) was obtained from the American Type Culture Collection (Manassas, VA, USA). Dulbecco’s modified Eagle’s medium (DMEM) mixed with 10% fetal bovine serum (FBS), 100 U/mL penicillin, and 100 µg/mL streptomycin was utilized for cell culture [15]. Cells were incubated at 37°C in a humidified atmosphere of 5% CO_2_.

### Exosome isolation and identification

Human serum exosomes were extracted with the ExoQuick Exosome Precipitation Solution (SBI System Biosciences, Mountain View, CA, USA), following the manufacturer’s protocol [16]. In brief, serum samples were centrifuged for 15 min at 3000 *g*. The ExoQuick Exosome Precipitation Solution was added to cell supernatants for 30 min at 4°C, centrifuged for 70 min at 10,000 *g*, and then decanted. Next, 100 µL of sterile 1× phosphate-buffered saline (PBS) was utilized for resuspension of the collected exosome sediments.

Exosomes from cells were isolated from 20 mL of culture media (1 × 10^7^ cells), based on protocols published by the manufacturer [17]. Briefly, cell medium was centrifuged for 10 min at 800 *g*, then for 30 min at 12,000 *g* to remove any cell debris. Next, the supernatant underwent another centrifugation at 100,000 *g* for 2 h to collect the precipitate. The exosome sediments were resuspended in 100 µL of PBS.

As described previously [18], transmission electron microscopy (TEM) was used to detect the size and form of the isolated exosomes, with the total exosome number determined using nanoparticle tracking analysis (NTA). Additionally, Western blotting was used to evaluate exosomal protein marker enrichment in exosomes.

For uptake studies, purified exosomes were labeled using the PKH26 labeling kit (Sigma-Aldrich, St. Louis, MO, USA) in compliance with instructions stipulated by the manufacturer. In brief, exosomes were resuspended in diluent C (containing PKH26 dye) and incubated for about 4–6 min. Labeled exosomes were then harvested and resuspended in serum-free medium, followed by co-culture with PTC cells for 24 h [19]. Next, slides were stained with 4ʹ,6-diamidino-2-phenylindole for 5 min, and then visualized them under a confocal laser scanning microscope.

### Western blotting

Radioimmunoprecipitation assay lysis buffer was used for the extraction of total proteins in exosomes and cells. As described previously [20], protein separation was performed using sodium dodecyl sulfate-polyacrylamide gel electrophoresis prior to being immunoblotted onto polyvinylidene fluoride membranes. Membranes were blocked with 5% nonfat milk at room temperature for an hour before they were subjected to an overnight incubation at 4°C with the following primary antibodies: anti-CD63 (Abcam, Cambridge, MA, USA), anti-CD81 (Abcam), anti-glyceraldehyde-3-phosphate dehydrogenase (GAPDH) (Santa Cruz Biotechnology, Santa Cruz, CA, USA), anti-vimentin (Cell Signaling Technology), anti-N-cadherin (Cell Signaling Technology), and anti-E-cadherin (Cell Signaling Technology, Danvers, MA, USA) antibodies. Samples were then incubated with secondary antibodies for an additional hour. An enhanced chemiluminescence reagents (Merck Millipore, Darmstadt, Germany) was used to visualize the membranes.

### Quantitative reverse-transcription polymerase chain reaction (qRT-PCR)

TRIzol reagent (Invitrogen) was used to extract total RNA. Exosomal RNA was isolated using the exoRNeasy Midi Kit (Qiagen) in strict accordance with instructions provided by the manufacturer [13]. cDNA synthesis was performed with the PrimeScript RT Reagent Kit (TaKaRa). The SYBR Premix Ex Taq II (TaRaKa) and the PrimeScript RT Reagent Kit (TaKaRa) were used for qRT-PCR analysis of circ007293 and PAX6 mRNA expression. GAPDH was used as the endogenous control. Mir-X miRNA First-Strand Synthesis Kit (TaKaRa) and SYBR Premix Ex Taq II (TaKaRa) were used for the for analysis of miR-653-5p expression. U6 was used as an endogenous control. The 2^–ΔΔCt^ method was used to evaluate relative gene expression [21]. The primers used in our study were listed as follows: circ007293 (F: 5ʹ-TGCCGGAAGAGGGGTTCCATG-3ʹ; R: 5ʹ-GCGAAGATTCTGCCCATCATGT-3ʹ), miR-653-5p (F: 5ʹ-GTGTTGAAACAATCTCTACTG-3ʹ; R: 5ʹ-GAACATGTCTGCGTATCTC-3ʹ), PAX6 (F: 5ʹ- TCTTTGCTTGGGAAATCCG-3ʹ; R: 5ʹ- CTGCCCGTTCAACATCCTTAG-3ʹ), and GAPDH (F: 5ʹ-TATGATGATATCAAGAGGGTAGT-3ʹ; R: 5ʹ-TGTATCCAAACTCATTGTCATAC-3ʹ).

### Cell transfection

The plasmid pcDNA3.1-CMV-circ007293 (oe-circ007293) was designed and created by GenePharma (Shanghai, China). Small interfering RNAs (siRNAs) against circ007293 (si-circ007293) and negative control (si-NC), miR-653-5p mimic (miR-653-5p) and miR-negative control (miR-NC), as well as siRNAs against PAX6 (si-PAX6) and negative control (si-Ctrl) were obtained from RiboBio (Guangzhou, China). Lipofectamine 2000 (Invitrogen) was used to transfect cells in accordance with protocols stipulated by the manufacturer [22].

### Cell counting kit-8 (CCK-8) assay

Cell viability was determined employing CCK-8 assay [12]. TPC-1 and KTC-1 cells (3 × 10^3^ cells/well) treated with or without exosomes (10 µg/mL) were cultured in 96-well plates for 24, 48, or 72 h. Next, 10 µL of CCK-8 reagent was added to each well, followed by a one-hour incubation period at 37°C. The absorbance at 450 nm was determined using a microplate reader (BioTek, Winooski, VT, USA).

### Wound healing assay

TPC-1 and KTC-1 cells (2 × 10^5^ cells/well) were seeded in 6-well plates. A scratch wound was used to scratch the surface of confluent cell layers. A serum-free medium containing 1 mM mitomycin was used to suppress cell division. At 0 and 24 h post scratching, the scratches were imaged with an inverted microscope (Olympus, Tokyo, Japan).

## Transwell assay

PTC cell invasion was quantified using a transwell chamber (8 µm pore size; Corning, NY, USA) [23]. The upper chambers were coated with Matrigel and used to house seeded with TPC-1 and KTC-1 cells. The lower chambers contained RPMI 1640 media supplemented with 10% FBS (500 µL). The system was incubated for 24 h before upper chamber was wiped using a cotton swab. Next, the invaded cells were fixed in 4% paraformaldehyde, treated using 0.5% crystal violet, and quantified using an inverted microscope (Olympus, Tokyo, Japan).

### Subcellular fractionation

The PARIS Kit (Invitrogen) was used for the isolation of nuclear and cytoplasmic fractions in accordance with protocols included in the kit [11]. Briefly, TPC-1 and KTC-1 cells were resuspended in fractionation buffer and centrifuged at 500 *g* for 5 min at 4°C. The cytoplasmic fractions were separated and placed in a fresh RNase-free tube. A cell fractionation buffer was then used to lyse the nuclear pellet. A lysis/binding solution was added to the nuclear lysate and cytoplasmic fractions before treatment with 100% ethanol. RNA isolation was used to divide the sample mixture. Isolated RNA was detected by qRT-PCR analysis with U6 and GAPDH acting as the nuclear and cytoplasmic controls, respectively.

### Dual-luciferase reporter assay

TPC-1 and KTC-1 cells (6 × 10^4^ cells/well) were incubated in 24-well plates for 24 h. A luciferase reporter vectors containing circ007293-miR-653-5p or PAX6 mRNA 3ʹUTR-miR-653-5p binding sequences (circ007293-WT or PAX6-WT) or mutant sequences (circ007293-MUT or PAX6-MUT) and miR-653-5p mimic or miR-NC were co-transfected using Lipofectamine 2000 (Thermo Fisher Scientific). Luciferase activity was assessed with the Dual Luciferase Reporter Assay System (Promega, Madison, WI, USA) for 48 h following instructions stipulated by the manufacturer [24].

### Statistical analysis

All data are presented as mean ± standard deviation (SD) and analyzed with the SPSS software (version 17.0; SPSS, Chicago, IL, USA). Statistical analysis was performed using Student’s *t*-test or one-way analysis of variance (ANOVA), followed by Turkey’s multiple-comparison test. Statistical significance was set at p < 0.05.

## Results

### Circ007293 was enriched in extracted exosomes from PTC patient serum and PTC cells

**Figure 1.**
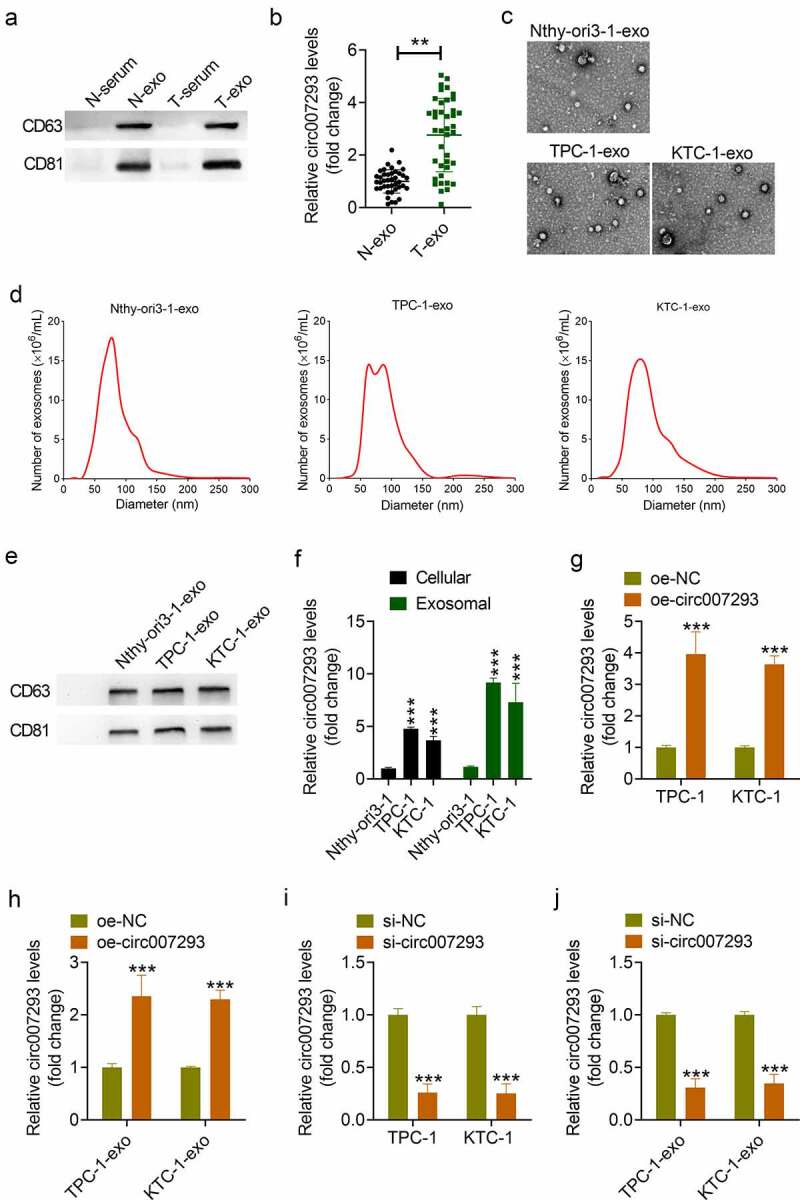
**Circ007293 was enriched in the exosomes from PTC patient serum and PTC cell lines**. (a) Western blotting results of CD63 and CD81 expression in serum of PTC patients (T-serun; n = 40), serum of healthy controls (N-serum; n = 40), exosomes derived from the serum of PTC patients (T-exo; n = 40) or healthy controls (N-exo; n = 40). (b) The expression of circ007293 in T-exo or N-exo was analyzed using qRT-PCR. (c-e) TEM, NTA, and Western blotting were performed to confirm exosomes derived from Nthy-ori3-1 (Nthy-ori3-1-exo), TPC-1 (TPC-1-exo), or KTC-1 (KTC-1-exo) cells. (f) The expression of circ007293 in different PTC cell lines and exosomes derived from different cells was analyzed using qRT-PCR. (g) The expression of circ007293 in TPC-1 and KTC-1 cells transfected with oe-circ007293 or oe-NC was determined by qRT-PCR. (h) The expression of circ007293 in exosomes derived from TPC-1 and KTC-1 cells transfected with oe-circ007293 or oe-NC was analyzed using qRT-PCR. (i) The expression of circ007293 in TPC-1 and KTC-1 cells transfected with si-circ007293 or si-NC was analyzed using qRT-PCR. (j) The expression of circ007293 in exosomes derived from TPC-1 and KTC-1 cells transfected with si-circ007293 or si-NC was determined using qRT-PCR. **p < 0.01, and ***p < 0.001 compared to the control group

A previous study reported upregulated circ007293 expression in exosomes extracted from PTC patient serum [25]. To confirm the enrichment of circ007293 in exosomes from PTC serum and PTC cells, exosomes from the serum of 40 PTC patients (T-exo) and 40 healthy controls (N-exo) were isolated and confirmed (Figure 1(a)), and circ007293 expression in serum exosomes from PTC patients and healthy controls was evaluated in this study. In accordance with previous findings, our findings demonstrated a marked in circ007293 levels in T-exo in contrast to N-exo (Figure 1(b)). In addition, the level of serum exosomal circ007293 was correlated with clinical stage and lymph node metastasis in PTC patients (Table 1). We also isolated exosomes from the culture medium of human thyroid cell line (Nthy-ori3-1) and PTC cell lines (KTC-1 and TPC-1). Cell-derived exosomes were analyzed by Western blotting, NTA, and TEM. TEM results revealed the presence of round vesicles with diameters between 50 and 150 nm (Figure 1(c)). NTA uncovered that these vesicles had a similar size distribution among these groups (Figure 1(d)). Moreover, Western blot analysis showed that the exosomal markers, CD81 and CD63, were noted to be enriched in the vesicles (Figure 1(e)). Furthermore, qRT-PCR analysis allowed for the detection of circ007293 levels in cells and corresponding exosomes (Figure 1(f)). Results showed that circ007293 expression levels in the PTC cell lines, TPC-1 and KTC-1, were notably raised in contrast to that in Nthy-ori3-1 cells. Simultaneously, circ007293 expression in PTC cell-derived exosomes was also much higher in contrast to that in Nthy-ori3-1-derived exosomes. The expression of circ007293 in exosomes was also higher than that in cells. Next, either oe-circ007293 or si-circ007293 was transfected into TPC-1 and KTC-1 cells, and the corresponding exosomes (oe-circ007293-exo or si-circ007293-exo) were isolated and purified from the culture medium. Results showed that overexpression of circ007293 significantly increased circ007293 levels in PTC cells and the corresponding exosomes secreted by PTC cells (Figure 1(g,h)) while silencing of circ007293 exerted the opposite effects (Figure 1(i,j)). This suggested that altering the level of circ007293 in cells could influence the expression of exosomal circ007293. Hence, our data indicated that circ007293 was enriched in exosomes secreted by PTC cells and may exert its function in the form of exosomes.

**Table 1.** The correlation between serum exosomal circ-007293 and clinicopathological features in PTC patients

Characteristic	n	Low expression	High expression	*P*-value
Age (years)				0.356
< 45	18	10	8	
≥ 45	22	9	13	
Gender				0.973
Male	14	6	8	
Female	26	11	15	
TNM				0.028
I–II	19	12	7	
III–IV	21	6	15	
Lymph node metastasis				0.049
Yes	17	5	12	
No	23	14	9	

### PTC cell-derived exosomal circ007293 enhanced proliferation, migration, invasion, and EMT in PTC cells

**Figure 2.**
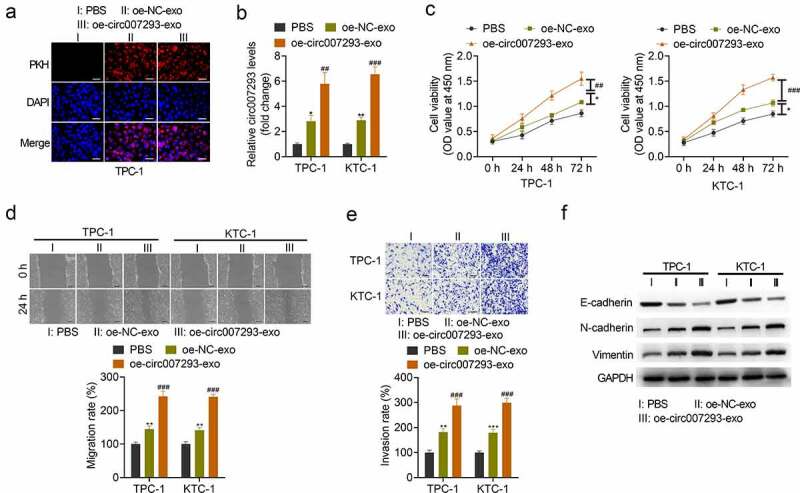
**PTC cell-derived exosomal circ007293 promoted PTC cell proliferation, migration, invasion, and EMT**. (a) Exosome uptake was assessed to confirm the uptake of PKH26-labeled oe-circ007293-exo (red) into recipient TPC-1 and KTC-1 cells. Magnification: ×200; scale bar: 50 μM. (b) Both oe-NC-exo and oe-circ007293-exo treatment increased the expression of circ007293 in TPC-1 and KTC-1 cells. (c) The viability of TPC-1 and KTC-1 cells co-cultured with oe-NC-exo or oe-circ007293-exo was evaluated using CCK-8 assay. (d) The migration of TPC-1 and KTC-1 cells co-cultured with oe-NC-exo or oe-circ007293-exo was analyzed using wound healing assay. Magnification: ×100; scale bar: 100 μM. (e) The invasion of TPC-1 and KTC-1 cells co-cultured with oe-NC-exo or oe-circ007293-exo was analyzed using transwell assay. Magnification: ×200; scale bar: 50 μM. (f) The levels of EMT protein markers (E-cadherin, N-cadherin, and vimentin) in TPC-1 and KTC-1 cells co-cultured with oe-NC-exo or oe-circ007293-exo were analyzed using Western blotting. *p < 0.05 and **p < 0.01 compared to the PBS group; ^##^p < 0.01 and ^###^p < 0.001 compared to the oe-NC-exo group

To confirm whether oe-circ007293-exo could metastasize in recipient cells, exosomes were labeled with PKH26 and then co-cultured with TPC-1 or KTC-1 cells for 24 h. As indicated by fluorescence microscopy, uptake of TPC-1- and KTC-1-derived exosomes was observed in the cytoplasm of PTC cells (Figure 2(a)). Similarly, circ007293 expression in KTC-1 and TPC-1 cells co-cultured with exosomes derived from PTC cells transfected with oe-NC (oe-NC-exo) and exosomes derived from circ007293 overexpressing PTC cells (oe-circ007293-exo) was markedly higher than that in KTC-1 and TPC-1 cells co-cultured with PBS. Additionally, the expression of circ007293 in PTC cells co-cultured with oe-circ007293 was significantly higher than that in PTC cells co-cultured with oe-NC-exo (Figure 2(b)).

Next, we evaluated the impact of exosomal circ007293 on the malignant phenotype of PTC cells. CCK-8 assay revealed that transfection with the PTC cell-derived exosomes, oe-NC-exo and oe-circ007293-exo, significantly increased the viability of PTC cell lines (Figure 2(c)). Wound healing and transwell assays confirmed that oe-circ007293-exo and oe-NC-exo treatment facilitated TPC-1 and KTC-1 cell migration and invasion (Figure 2(d,e)).

EMT is a critical facilitator of tumor metastasis and is characterized by upregulated expression of mesenchymal markers (e.g., N-cadherin, vimentin) and downregulated expression of epithelial markers (e.g., E-cadherin) [26]. To determine whether exosomal circ007293 affects the EMT process of PTC cells, the expression of E-cadherin, N-cadherin, and vimentin was detected in PTC cells co-cultured with oe-circ007293-exo or oe-NC-exo. Western blot analysis revealed that transfection with oe-circ007293-exo or oe-NC-exo downregulated the expression of E-cadherin, but N-cadherin and vimentin expressions in PTC cells (Figure 2(f)). Moreover, treatment with exosomal circ007293 enhanced the promoting effects of PTC cell-derived exosomes on PTC cell proliferation, migration, invasion, and EMT (Figure 2(b-f)). Collectively, our findings highlight that the effects of PTC cell-derived exosomes on PTC cell migration, invasion, and EMT might be mediated by circ007293.

### Knockdown of circ007293 suppressed the malignant phenotype of PTC cells

**Figure 3.**
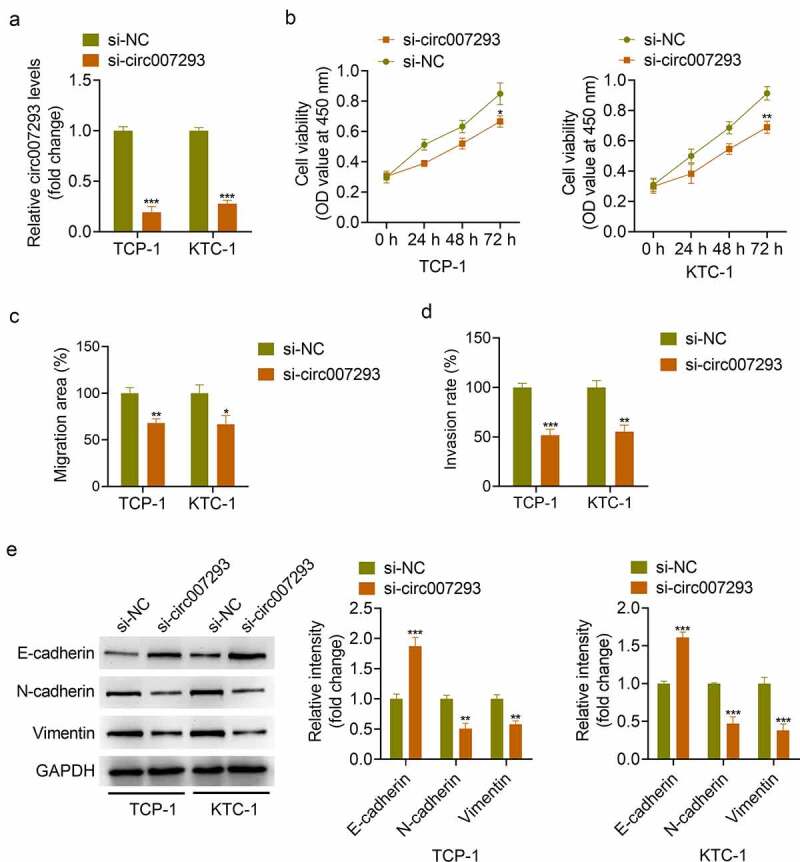
**Knockdown of circ007293 suppressed the malignant phenotype of PTC cells**. (a) circ007293 expression in TCP-1 and KTC-1 cells transfected with si-circ007293 or si-NC was determined using qRT-PCR. (b) The viability of TPC-1 and KTC-1 cells transfected with si-circ007293 or si-NC was evaluated using CCK-8 assay. (c) The migration of TPC-1 and KTC-1 cells transfected with si-circ007293 or si-NC was analyzed using wound healing assay. (d) The invasion of TPC-1 and KTC-1 cells transfected with si-circ007293 or si-NC was analyzed using transwell assay. (e) The expression levels of EMT protein markers (E-cadherin, N-cadherin, and vimentin) in TPC-1 and KTC-1 cells transfected with si-circ007293 or si-NC were determined using Western blotting. *p < 0.05, **p < 0.01, and ***p < 0.001 compared to the si-NC group

To examine whether circ007293 affected biological processes in PTC cells, TPC-1 and KTC-1 cells were transfected with si-circ007293 or si-NC. qRT-PCR results confirmed that si-circ007293 transfection significantly decreased circ007293 expression in PTC cells (Figure 3(a)). CCK-8 assay showed that circ007293 silencing markedly inhibited PTC cell proliferation (Figure 3(b)). Additionally, wound healing and transwell assays demonstrated that circ007293 silencing suppressed PTC cell migration and invasion (Figure 3(c,d)). Moreover, Western blot analysis showed that silencing of circ007293 inhibited EMT, as indicated by suppressed N-cadherin and vimentin expressions and increased E-cadherin expression (Figure 3(e)). These results indicated that knockdown of circ007293 suppressed the proliferation, migration, invasion and EMT of PTC cells.

### Circ007293 facilitated PAX6 expression by acting as a competing endogenous RNA (ceRNA) for miR-653-5p

**Figure 4.**
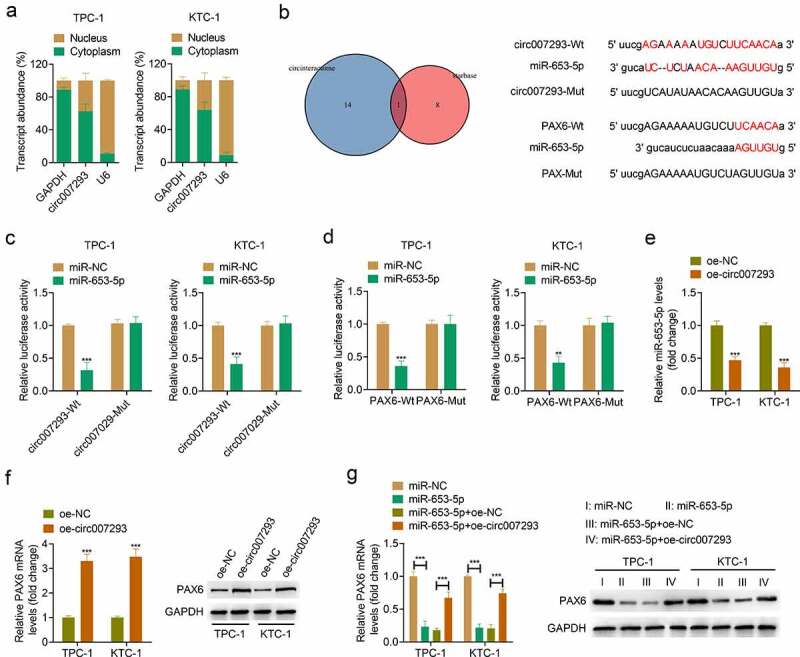
**Circ007293 promoted PAX6 expression through acting as a ceRNA for miR-653-5p**. (a) Subcellular fractionation for circ007293 in the nucleus and cytoplasm of TPC-1 and KTC-1 cells. (b) Left: Venn diagram showing miR-653-5p as the potential circ007293-targeting miRNA in the prediction results from Circinteractome and starBase. Right: Predicted binding sites of circ007293 and miR-653-5p, and miR-653-5p and PAX6 mRNA 3ʹUTR. (c) Evaluation of luciferase activity in TPC-1 and KTC-1 cells co-transfected with circ007293-WT or MUT reporter vectors and miR-653-5p or miR-NC. (d) Evaluation of luciferase activity in TPC-1 and KTC-1 cells co-transfected with PAX6-WT or MUT reporter vectors and miR-653-5p or miR-NC. (e) The expression of miR-653-5p in TPC-1 and KTC-1 cells transfected with oe-circ007293 or oe-NC was analyzed using qRT-PCR. (f) The mRNA and protein expression levels of PAX6 in TPC-1 and KTC-1 cells transfected with oe-circ007293 or oe-NC were determined by qRT-PCR and Western blotting, respectively. (g) The mRNA and protein expression levles of PAX6 in TPC-1 and KTC-1 cells co-transfected with miR-653-5p mimic and oe-circ007293 were analyzed using qRT-PCR and Western blotting, respectively. **p < 0.01 and ***p < 0.001

To further elucidate the molecular mechanism by which circ007293 affects the progression of PTC, the distribution of circ007293 in PTC cell nuclei and cytoplasm was determined using subcellular fractionation. Our experiments revealed that circ007293 was primarily enriched in PTC cell cytoplasm (Figure 4(a)). Subsequently, we used the online bioinformatics databases, Circinteractome, and starBase, to predict miRNAs that may be complementary to circ007293. A Venn diagram was used to visualize the intersection of prediction results of Circinteractome and starBase, revealing that miR-653-5p functioned as a potential target miRNA of circ007293 (Figure 4(b)). Moreover, through miRTarBase, starBase, and miRDB analysis, we found that miR-653-5p could complement the 3ʹUTR of PAX6, suggesting that PAX6 may be targeted by miR-653-5p (Figure 4(b)). Subsequently, a luciferase reporter assay enabled us to assess the association between circ007293, miR-653-5p, and PAX6. The upregulation of miR-653-5p was found in TPC-1 and KTC-1 cells transfected with miR-653-5p mimic compared with that transfected with miR-NC (supplementary Figure 1a). While, the downregulation of PAX6 was discovered in TPC-1 and KTC-1 cells transfected with si-PAX6 compared with cells transfected with si-Ctrl (supplementary Figures 1B and 1 C). The experiments demonstrated that transfection with miR-653-5p mimic downregulated the relative luciferase activity of circ007293-WT vectors without altering the luciferase activity of circ007293-MUT vectors (Figure 4(c)). Simultaneously, luciferase activity was markedly decreased in PTC cells co-transfected with PAX6-WT and miR-653-5p mimic but remained unchanged in cells co-transfected with PAX6-MUT vectors and miR-653-5p mimic (Figure 4(d)). Besides, qRT-PCR and Western blotting were used to assess the miR-653-5p and PAX6 expressions in TPC-1 and KTC-1 cells transfected with oe-circ007293. We found that circ007293 overexpression downregulated miR-653-5p expressions in PTC cells (Figure 4(e)) but upregulated the mRNA and protein expressions of PAX6 (Figure 4(f)). Furthermore, miR-653-5p mimic and circ007293 overexpression vectors were co-transfected into TPC-1 and KTC-1 cells. We found that miR-653-5p upregulation downregulated PAX6 expression in PTC cells, but co-transfection with circ007293 overexpression vector attenuated this effect (Figure 4(g)). These data confirmed that circ007293 acted as an miR-653-5p sponge to facilitate PAX6 expression in PTC cells.

### Exosomal circ007293 promoted PCT cell malignant phenotype via the miR-653-5p/PAX6 axis

**Figure 5.**
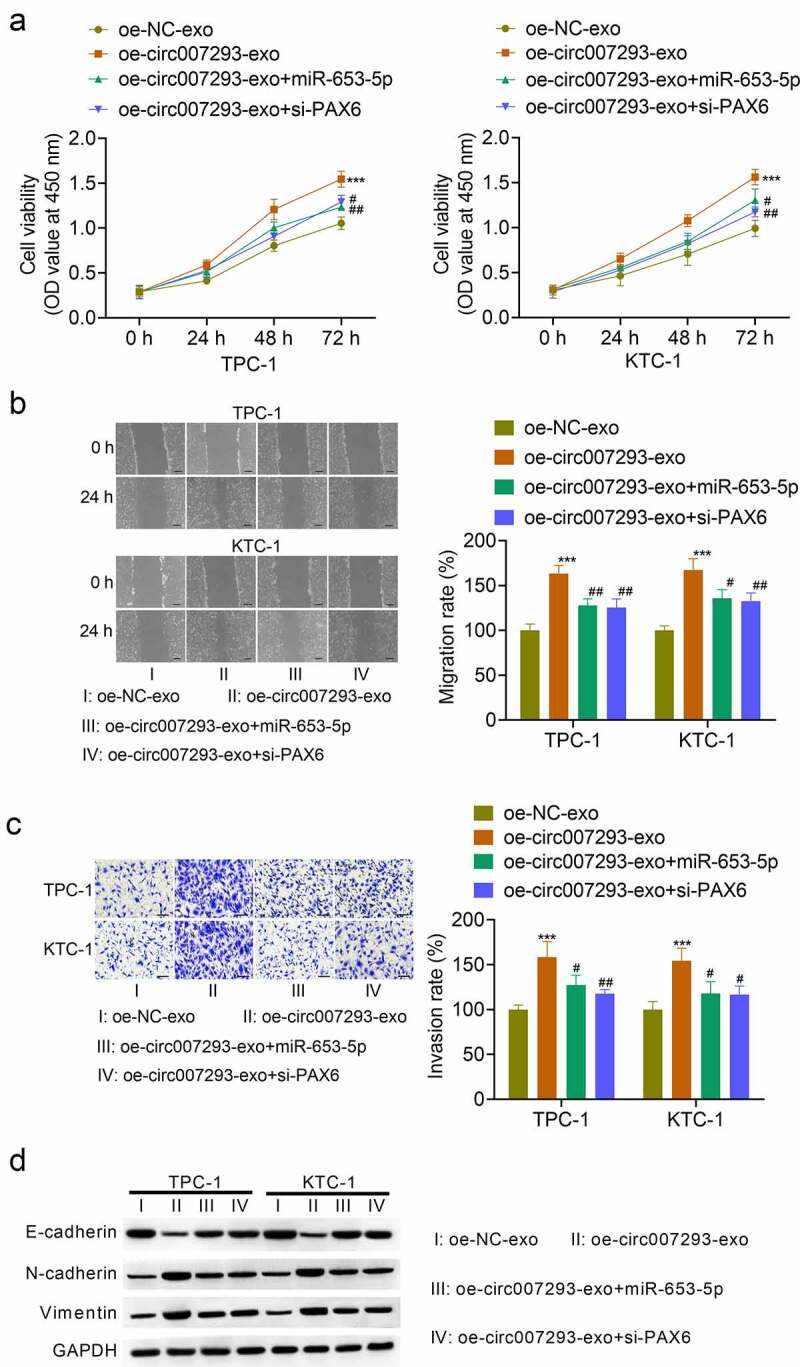
**Exosomal circ007293 promoted the malignant phenotype of PCT cells via the miR-653-5p/PAX6 axis**. TPC-1 and KTC-1 cells transfected with miR-653-5p mimic or si-PAX6 were treated with oe-circ007293-exo or oe-NC-exo. (a) The viability of TPC-1 and KTC-1 cells was evaluated using CCK-8 assay. (b) The migration of TPC-1 and KTC-1 cells was analyzed using wound healing assay. Magnification: ×100; scale bar: 100 μM. (c) The invasion of TPC-1 and KTC-1 cells was analyzed using transwell assay. Magnification: ×200; scale bar: 50 μM. (d) The expression of E-cadherin, N-cadherin, and vimentin in TPC-1 and KTC-1 cells was determined by Western blot. ***p < 0.001 compared to the oe-NC-exo group; #p < 0.05 and ##p < 0.01 compared to the oe-circ007293-exo group

To confirm whether exosomal circ007293 regulated PTC cell proliferation and metastasis via the miR-653-5p/PAX6 axis, TPC-1 and KTC-1 cells were transfected with miR-653-5p mimic or si-PAX6, followed by treatment with oe-circ007293-exo. Transfection with miR-653-5p mimic notably enhanced PTC cell miR-653-5p expression, while transfection with si-PAX6 remarkably suppressed the mRNA and protein expression of PAX6 in PTC cells (data not shown). As expected, exosomal circ007293-induced promotion of cell viability attenuated by transfection with miR-653-5p mimic and si-PAX6 (Figure 5(a)). Consistently, miR-653-5p overexpression or PAX6 silencing in PTC cells reversed the effects of exosomal circ007293 on PTC cell migration and invasion to a certain extent (Figure 5(b,c)). In addition, upregulation of miR-653-5p or silencing of PAX6 could reversed the effect of exosome-derived circ-007293 on the expression of EMT protein markers (E-cadherin, N-cadherin, and vimentin) in TPC-1 and KTC-1 cells (Figure 5(d)). Collectively, our data indicate that exosomal circ007293 regulates the malignant phenotype of PTC cells through the miR-653-5p/PAX6 axis.

## Discussion

Previous studies have confirmed aberrantly expressed circRNAs across various human diseases, which includes thyroid cancer. More than one circRNAs are likely to work in tandem to regulate tumor immunosuppression, proliferation, migration, invasion, and chemoresistance [27,28]. Notably, circRNAs are enriched in exosomes and are transferable to target cells [29]. In the current study, we demonstrated that exosomal circ007293 could be delivered to PTC cells and took part in modulating PTC cell malignant phenotypes. Mechanistically, exosomal circ007293 acted as an miR-653-5p sponge by inhibiting miR-653-5p activity, thereby increasing the expression of PAX6 in PTC cells and promoting tumor cell proliferation, metastasis, and EMT.

Several studies have confirmed that cell-to-cell communication plays a vital role in promoting cancer progression [9,30]. Exosomes, which play a role in paracrine signaling in different cell types, are implicated in intercellular communication by carrying functional information [9]. Generally, exosomes are representative of the malignant characteristics of donor cells and transmit oncogenic signals to recipient cells, thus promoting cancer progression [31]. Exosomes are composed of specific proteins, RNA, ncRNA (including circRNA, long non-coding RNA, and miRNA), and lipids. Recent reports have confirmed that miRNAs and circRNAs derived from exosomes can mediate gene-based communication between cells [32]. In this study, we utilized TEM and NTA to determine the characteristics of exosomes in PTC patient-derived serum and PTC cell culture media. Notably, circ007293 was highly enriched in serum- and cell-derived exosomes. Additionally, both gain- or loss-of-function assays confirmed that altering circ007293 expressions in cells influenced the amount of exosomal circ007293. Functionally, an increase in exosomal circ007293 levels enhanced the impact of exosomes on cell invasion, migration, proliferation, and EMT, suggesting that circ007293 may participate in cellular communication through the delivery of exosomes. The role of circRNAs in PTC carcinogenesis has been extensively studied. Previously, circ102171 was shown to promote PTC progression via β-catenin-interacting protein 1-dependent activation of the β-catenin pathway [33]. Yao et al. [34] discovered that the circNEK6/miR-370-3p axis was implicated in PTC progression and initiation. Liu et al. [35] also demonstrated that hsa_circ_0060060 enhances the cisplatin resistance of PTC cells through the regulation of autophagy. Moreover, circ_0062389 could sponge miR-1179 to increase the expression of high mobility group box 1, and then promote the proliferation, migration, and EMT process of PTC cells [12]. In the current study, circ007293 promoted PTC cell invasion, migration, proliferation, and EMT via the exosome-mediated transferred mechanism, indicating the carcinogenic properties of circ007293 in PTC cells. The above findings indicate that circ007293 is enriched in exosomes secreted by PTC cells and may exert its carcinogenic effect in the form of exosomes.

In view of the inclusion of conserved miRNAs target sites, circRNAs can competitively suppress miRNAs regulation via their effects as a miRNA sponge on downstream target genes [4]. In order to explore whether circ007293 participated in PTC in a similar way, we confirmed miR-653-5p as one of the target miRNAs of circ007293 using Circinteractome and starBase. As the most widely studied ncRNA, miRNAs have been proven to act as oncogenes or tumor suppressor genes [36]. For example, a previous study that demonstrated that prostate cancer cell invasion and proliferation were suppressed by miR-653-5p inhibition; a phenomenon thought to occur as a result of downregulation of SRY-box 30 expression [25]. It is well known miR-653-5p may target chromosome 11 open reading frame 30, and miR-653-5p acts as a tumor suppressor gene in cervical cancer [37]. Besides, Han et al. [38] confirmed that circHIPK3 promotes the invasion of non-small cell lung cancer cells by acting as an miR-653-5p sponge. Here, our findings demonstrate that circ007293 downregulated miR-653-5p expression in PTC cells. In addition, rescue experiments suggested that exosomal circ007293-induced promotion of the malignant behavior of PTC cells appeared to be facilitated through miR-653-5p inhibition. Therefore, circ007293 could competitively bind miR-653-5p to induce EMT, thus augmenting PTC cell invasion and metastasis.

Canonically, miRNAs can delay translation or induce the degradation of target mRNA by pairing with its 3ʹ untranslated region [39]. Several target genes of miR-653-5p, such as signal transducer and activator of transcription 2 in neuroblastoma [40] as well as retinoic acid induced 14 in melanoma [41], have been identified. Here, through miRTarBase, starBase, and miRDB analyses, we discovered that PAX6 might potentially target miR-653-5p, which was further confirmed by dual luciferase reporter assay. PAX6 belongs to the PAX family and is a vital transcription factor in a variety of biological processes [42]. Other investigations have also supported PAX6 as an oncogene and have characterized its overexpression across numerous types of human cancers [43,44]. Moreover, its cancer-promoting effects can be regulated by miRNAs [43,45,46]. Here, our findings confirmed the inhibitory impact of miR-653-5p on PAX6 expression in PTC cells. This effect was reversed by upregulating circ007293 expression. Consistently, PAX6 knockdown abolished the cancer-promoting effects of exosomal circ007293 in PTC cells *in vitro*. Collectively, exosomal circ007293 could regulate the malignant phenotype of PTC cells by targeting the miR-653-5p/PAX6 axis.

The current study has certain limitations. First, instead of RNA sequencing, qRT-PCR was utilized for the detection of exosomal circRNAs that were differentially expressed between PTC patients and healthy controls. Secondly, analyzing the diagnostic and prognostic values of exosomal circ007293 in the PTC population using a larger sample size may be of higher clinical significance. Moreover, the biological functions of exosomal circ007293 *in vivo* were not investigated, and should be explored in future studies.

## Conclusion

In summary, our findings implicated that exosomal circ007293 in regulating PTC cell invasion, migration, proliferation, and EMT. Moreover, our study also demonstrated that circ007293 could induce PAX6 expression by sponging miR-653-5p. These data provide further insights on the mechanism underlying exosomal circ007293-mediated PTC progression, which may help in the development of novel therapeutic strategies.

## Supplementary Material

Supplemental Material

## Data Availability

**A**ll data generated or analyzed during this study are included in this published article and its additional files.
