# Supplementary figures and images for: Exosomal circular RNA hsa_circ_007293 promotes proliferation, migration, invasion, and epithelial–mesenchymal transition of papillary thyroid carcinoma cells through regulation of the microRNA-653-5p/paired box 6 axis
Source: Bioengineered. 2021 Dec 6;12(2):10136–49. doi: 10.1080/21655979.2021.2000745 (PMC8809932; doi:10.1080/21655979.2021.2000745)

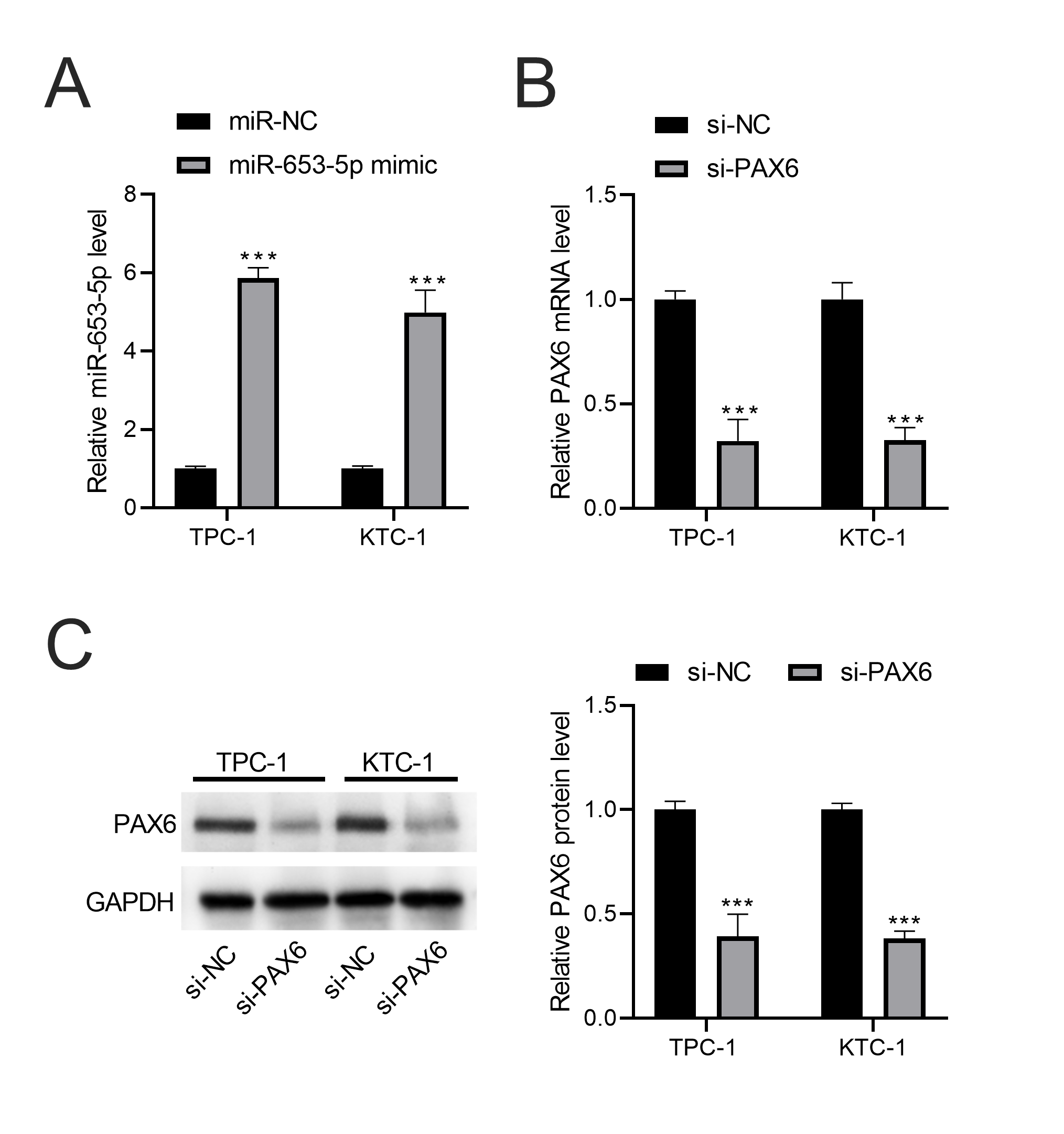

Supplement: Supplemental Material [file KBIE_A_2000745_SM6310.tif]
